# Aspartame and cancer – new evidence for causation

**DOI:** 10.1186/s12940-021-00725-y

**Published:** 2021-04-12

**Authors:** Philip J. Landrigan, Kurt Straif

**Affiliations:** 1 Program for Global Public Health and the Common Good, Boston College, 140 Commonwealth Avenue / Higgins Hall, Suite 648, Chestnut Hill, MA 02467 USA; 2 Global Observatory on Pollution and Health, Boston College, 140 Commonwealth Avenue / Higgins Hall, Suite 648, Chestnut Hill, MA 02467 USA; 3 Schiller Institute for Integrated Science and Society, Boston College, 140 Commonwealth Avenue / Higgins Hall, Suite 648, Chestnut Hill, MA 02467 USA; 4 Boston College, Chestnut Hill, MA USA; 5 ISGlobal, Barcelona, Spain

**Keywords:** Aspartame, Cancer, Artificial sweetener, Tumors, Pulmonary lymphoma, Leukemia, Carcinogenicity, Ramazzini institute

## Abstract

**Background:**

Aspartame is one of the world’s most widely used artificial sweeteners and is an ingredient in more than 5000 food products globally. A particularly important use is in low-calorie beverages consumed by children and pregnant women.

The Ramazzini Institute (RI) reported in 2006 and 2007 that aspartame causes dose-related increases in malignant tumors in multiple organs in rats and mice. Increased cancer risk was seen even at low exposure levels approaching the Acceptable Daily Intake (ADI). Prenatal exposures caused increased malignancies in rodent offspring at lower doses than in adults.

These findings generated intense controversy focused on the accuracy of RI’s diagnoses of hematopoietic and lymphoid tissue tumors (HLTs). Critics made the claim that pulmonary lesions observed in aspartame-exposed animals were inflammatory lesions caused by *Mycoplasma* infection rather than malignant neoplasms.

**Methods:**

To address this question, RI subjected all HLTs from aspartame-exposed animals to immunohistochemical analysis using a battery of markers and to morphological reassessment using the most recent Internationally Harmonized Nomenclature and Diagnostic (INHAND) criteria.

**Findings:**

This immunohistochemical and morphological re-evaluation confirmed the original diagnoses of malignancy in 92.3% of cases. Six lesions originally diagnosed as lymphoma (8% of all HLTs) were reclassified: 3 to lymphoid hyperplasia, and 3 to chronic inflammation with fibrosis. There was no evidence of *Mycoplasma* infection.

**Interpretation:**

These new findings confirm that aspartame is a chemical carcinogen in rodents. They confirm the very worrisome finding that prenatal exposure to aspartame increases cancer risk in rodent offspring. They validate the conclusions of the original RI studies.

These findings are of great importance for public health. In light of them, we encourage all national and international public health agencies to urgently reexamine their assessments of aspartame’s health risks - especially the risks of prenatal and early postnatal exposures. We call upon food agencies to reassess Acceptable Daily Intake (ADI) levels for aspartame. We note that an Advisory Group to the International Agency for Research on Cancer has recommended high-priority reevaluation of aspartame’s carcinogenicity to humans.

## Introduction

For decades, controversy has surrounded the question of whether the artificial sweetener, aspartame can cause cancer.

Aspartame was first manufactured in 1965. In 1981, following cursory assessment of its safety and toxicity [1], aspartame was approved by the U.S. Food and Drug Administration for use in foods [2]. Today with an annual production of 3000–5000 metric tons, aspartame is one the world’s most widely used artificial sweeteners. It is an ingredient in more than 5000 food and beverage products including cereals, chewing gum, yogurt, pharmaceuticals, and instant coffee. A particularly important use in the United States is in the manufacture of low-calorie beverages that are extensively consumed by children and pregnant women [3].

### The Ramazzini Institute studies of aspartame

In 1997, in response to rising concerns about the safety of aspartame, the Ramazzini Institute (RI), an independent, not-for-profit research laboratory in Bologna, Italy initiated a series of large-scale toxicological studies of the possible carcinogenicity of aspartame. In the first of these studies (BT 6008), aspartame was administered to Sprague-Dawley rats in their feed at seven dose levels ranging from 0 to 100,000 ppm (ppm) throughout their lives beginning at 8 weeks of age [4]. The second study (BT 6009) used the three lowest doses of the first study, but began dosing prenatally, thus resulting in exposures to fetal rat pups in utero [5]. The third study (BT 6010) was performed on Swiss mice, used five dose levels, and again began dosing prenatally [6]. In total, 2270 Sprague-Dawley rats and 852 Swiss mice were included in these three studies.

The main finding in these RI studies was that aspartame caused increased incidence of malignant tumors in multiple organs in rodents. Increases were seen in hematopoietic and lymphoid tissue tumors (HLTs) in animals of both sexes, carcinomas of the renal pelvis and the ureter in females, mammary cancers in females, and malignant schwannomas of the peripheral nerves in males. Positive dose-response relationships were observed, in which the highest incidence of malignancies was seen in the animals exposed to the highest levels of aspartame [4–7].

Increased incidence of malignant tumors was seen even in animals exposed to relatively low doses of aspartame – exposures close to the current Acceptable Daily Intake (ADI) levels of 40 mg/Kg body weight in the European Union [8, 9] and 50 mg/Kg body weight in the United States [10].

**Fig. 1:**
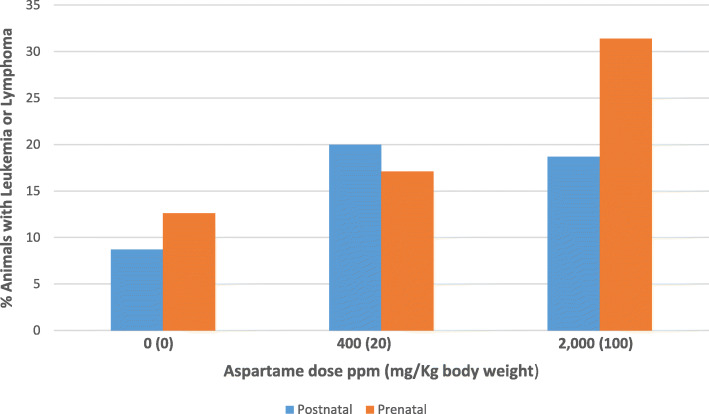
Lymphoma/Leukemia Incidence in Female Sprague-Dawley Rats Exposed to Aspartame. Comparison of Prenatal v. Postnatal Exposure

Prenatal exposures of rat pups to aspartame in utero produced dose-related increases in malignancies at lower exposure levels and with shorter latency periods than body weight equivalent exposures in adults [5] (Fig. 1). This finding indicates that aspartame may initiate carcinogenesis in utero*.* It is consistent with a large body of literature indicating that young animals, especially in the fetal period, are more sensitive than older animals to a range of chemical and physical carcinogens [11].

Three unique features of the RI’s toxicological testing protocol distinguish it from most other carcinogenesis bioassays [7, 12]:

1. Large numbers of animals are used, thus increasing statistical power to detect increases in cancer incidence;
2. Animals are maintained and followed across their entire lifetimes to natural death. This design replicates the human experience, in which approximately 80% of all cancers are diagnosed beyond the age of 60 years [13]. It enables the RI to detect malignancies that are missed by many other rodent bioassays that truncate follow-up and sacrifice their experimental animals at 104 weeks (or earlier) – often before the chemical under examination has had an opportunity to express its carcinogenic potential, and before many chemically induced malignancies have become evident [7, 14];
3. Systematic histopathological analyses are undertaken in all organs and tissues, and not merely in a subset.

### The controversy

Publication of the RI findings on the carcinogenicity of aspartame generated intense controversy [15]. At the heart of this debate were doubts raised about the accuracy of the RI’s histopathological diagnoses - in particular the RI’s diagnoses of pulmonary lymphomas and leukemias – in animals exposed to aspartame [14, 16].

The European Food Safety Agency (EFSA) made the unsubstantiated claim that the RI’s animal colony was poorly managed and that the experimental animals were subject to uncontrolled infections [8, 9, 17]. Schoeb et al. speculated further that pulmonary lesions diagnosed as lymphomas and leukemias by RI might have been inflammatory lesions caused by *Mycoplasma pulmonis* infections [18].

Of note is that none of these explanations accounted for the strongly positive dose-response relationships between aspartame exposure level and cancer incidence observed in the RI studies or for the increased incidence of neoplasms in animals exposed in utero [19].

## Methods

### Resolution of the controversy

To address these issues, RI reexamined all lesions in Sprague-Dawley rats that had been diagnosed as hematopoietic and lymphoid tissue tumors (HLTs) in the experimental study that initiated aspartame dosing prenatally (BT 6009). Two state-of-the-art diagnostic techniques were employed:

- Immunohistochemical analyses. All lesions diagnosed as HLTs were subjected to immunohistochemical analysis to assess the clonality of the tissues. These analyses used the following battery of markers: Ki67, CD3, PAX5, CD20, CD68, TdT, CD45, CD14 and CD33.
  The premise underlying immunohistochemical analysis is that all cells in a hematologic or lymphoid malignancy are expected to be immunohistochemically identical - i.e., monoclonal - because they are all the direct descendants of a single transformed cell [14, 20]. By contrast, the inflammatory lymphocytes that respond to an infection are of diverse cellular origin and are therefore not immunohistochemically identical – i.e., polyclonal.
  Immunohistochemical analysis provides a powerful complement to morphological examination of tissues and improves diagnostic accuracy [14].
- Morphological reclassification. The morphological features of all lesions that had originally been diagnosed as lymphoma or leukemia were reexamined and reclassified according to the most recently updated INHAND criteria - the Internationally Harmonized Nomenclature And Diagnostic (INHAND) criteria for the pathological diagnosis of lesions in rats and mice [21].

## Results

Immunohistochemical analysis and morphological reclassification of all lesions originally diagnosed as hematopoietic and lymphoid tissue tumors (HLTs) have now been completed [22]. This reassessment confirmed that 72 (92.3%) of the 78 lesions originally diagnosed as HLTs were malignant tumors and that another 3 (3.8%) were premalignant lesions. An additional three lesions were reclassified as chronic inflammation with fibrosis. There was no histological evidence of infection caused by *Mycoplasma pulmonis* or other microorganisms.

Statistical reanalysis based on these data reconfirmed the three main findings of the original RI study [5]:

1. There is a statistically significant increase in incidence of all hematolymphatic malignancies (*p* = 0.006), including significant increases in both lymphomas (*p* = 0.032) and leukemias (*p* = 0.031). in rodents exposed to aspartame;
2. There is a positive dose-response relationship between aspartame exposure and incidence of hematolymphatic malignancy; *and*
3. Prenatal exposures to aspartame produce dose-related increases in malignancies at lower exposure levels and with shorter latency than equivalent exposures in adults (Fig. 1).

## Implications for public health and Cancer prevention

The state-of-the-art reanalysis of the Ramazzini Institute data [22] confirms that aspartame is a chemical carcinogen in rodents. This reanalysis confirms that 92% of the lesions observed in experimental animals exposed to aspartame in the RI studies were indeed malignant. It rebuts the claim that infection by *Mycoplasma* or some other microorganism was responsible for the lesions. In short, this reanalysis provides powerful, validation of the original RI conclusions [5].

The finding of increased HLTs in animals exposed to aspartame at a dose of 100 mg/Kg body weight is of great concern. This is a relatively low-dose exposure - dangerously close to the current Acceptable Daily Intake (ADI) levels for aspartame established by regulatory agencies in the European Union [8, 9] and the United States [10]. In large populations, significant numbers of persons frequently consume food ingredients, aspartame included, at levels exceeding ADIs. Such exceedances are especially common among infants and young children because of their greater food intake per Kg body weight compared to adults and their unusual dietary patterns [11]. The implication of this finding is that current ADI levels for aspartame may be set too high and may not offer sufficient protection against cancer. ADI levels for aspartame need urgently to be reevaluated, especially as they apply to pregnant women and young children.

The RI reanalysis [22] documents the power of new technologies such as immunohistochemical analysis [14, 20] and harmonized diagnostic classification, such as the INHAND classification [21], to resolve diagnostic controversy. These state-of-the-art techniques improve accuracy of diagnoses of lymphoma and leukemia in rats. Going forward, standardized techniques such as these should routinely be incorporated into all toxicity assays, just as standard diagnostic criteria are now used for classification of hematolymphatic malignancies in humans [23].

The finding that prenatal exposure to aspartame increases incidence of leukemia and lymphoma in offspring in rodents is of grave concern (Fig. 1). Pregnant women and young children consume large quantities of foods and beverages sweetened with aspartame [24]. In the United States, pregnant women extensively consume aspartame-containing soft drinks to prevent weight gain during pregnancy. Fetal aspartame exposure is the inevitable consequence*.* These findings raise the possibility that aspartame may be a contributor to current increases in incidence of leukemia and other cancers in children [25].

National and international public health agencies need to take careful notice of these revalidated findings. Previous facile dismissals of the carcinogenicity of aspartame can no longer be sustained [8, 9, 17, 18]. Long experience documents that delay in acting on well-documented evidence of chemical carcinogenesis results in unnecessary disease and preventable death [26–28].

The findings presented here underscore the need for epidemiologic studies of cancer incidence in populations exposed to aspartame – especially children exposed to aspartame in utero. To date, only two epidemiologic studies have been conducted of aspartame-exposed populations. The first, a 2006 study conducted in a very large population of middle aged Americans by the US National Cancer Institute, showed no carcinogenic effect [29]. Although the population was large, this study used a relatively weak questionnaire instrument for assessing aspartame exposures and appears to have been subject to exposure misclassification. Moreover, reported exposures were generally low and the study was not designbed to assess the consequences of aspartame exposures in early life. A second epidemiological study conducted within the prospectively followed population of the Harvard Nurses Health Study carefully assessed exposures and reported a significantly elevated risk of non-Hodgkin lymphoma (NHL) in males who consumed one or more servings of soda per day [30]. There reappeared to be a positive exposure-response relationship between soda consumption and NHL risk. Additional, carefully conducted epidemiological studies of the potential of aspartame to cause cancer in humans are very much needed, with a particular focus on early-life exposures.

We call upon all national and international public health agencies to urgently reexamine their assessments of aspartame’s risks to health - especially the risks of prenatal exposure – in light of these newly revalidated findings from the Ramazzini Institute. This call reiterates a plea for such reexamination that was made by Ramazzini Institute scientists in 2014 [31]. We call upon food agencies in countries around the world to reassess Acceptable Daily Intake (ADI) levels for aspartame.

We note that the Advisory Group on Future Priorities for the International Agency for Research on Cancer’s Monographs Program has recently recommended that the potential carcinogenicity of aspartame to humans be evaluated with high priority within the next 2.5 years [32].

## References

[1] Huff J, Ladou J (2007). Aspartame bioassay findings portend human cancer hazards. Int J Occup Environ Health.

[2] FDA (Food and Drug Administration) (1981). Aspartame: Commissioner’s Final Decision. [Docket No. 75F-0355]. Fed Reg.

[3] Fitch C, Keim KS (2012). Position of the academy of nutrition and dietetics: use of nutritive and nonnutritive sweeteners. J Acad Nutr Diet.

[4] Soffritti M, Belpoggi F, Degli Esposti D, Lambertini L, Tibaldi E, Rigano A (2006). First experimental demonstration of the multipotential carcinogenic effects of aspartame administered in the feed to Sprague-Dawley rats. Environ Health Perspect.

[5] Soffritti M, Belpoggi F, Tibaldi E, Degli Esposti D, Lauriola M (2007). Life-span exposure to low doses of aspartame beginning during prenatal life increases Cancer effects in rats. Environ Health Perspect.

[6] Soffritti M, Belpoggi F, Manservigi M, Tibaldi E, Lauriola M, Falcioni L, Bua L (2010). Aspartame administered in feed, beginning prenatally through life span, induces cancers of the liver and lung in male Swiss mice. Am J Ind Med.

[7] Belpoggi F, Soffritti M, Padovani M, Degli Esposti D, Lauriola M, Minardi F (2006). Results of long-term carcinogenicity bioassay on Sprague-Dawley rats exposed to aspartame administered in feed. Ann NY Acad Sci.

[8] EFSA (European Food Safety Authority) (2009). Updated opinion on a request from the European Commission related to the 2nd ERF carcinogenicity study on aspartame, taking into consideration study data submitted by the Ramazzini foundation in February 2009. Scientific opinion of the panel on food additives and nutrient sources added to food. EFSA J.

[9] EFSA (European Food Safety Authority) (2013). Scientific Opinion on the re-evaluation of aspartame (E 951) as a food additive. EFSA J.

[10] FDA (Food and Drug Administration) (1996). Food additives permitted for direct addition to food for human consumption: Aspartame. Fed Regist.

[11] NRC (National Research Council) (1993). Pesticides in the Diets of Infants and Children.

[12] Huff J, Jacobson MF, Davis DL (2008). The limits of two-year bioassay exposure regimens for identifying chemical carcinogens. Environ Health Perspect.

[13] (GBD) Global Burden of Disease Collaborative Network (2018). Global Burden of Disease Study 2017 (GBD 2017).

[14] Gift JS, Caldwell JC, Jinot J, Evans MV, Cote I, Vandenberg JJ (2013). Scientific considerations for evaluating cancer bioassays conducted by the Ramazzini institute. Environ Health Perspect.

[15] Millstone EP, Dawson E (2019). EFSA’s toxicological assessment of aspartame: was it even-handedly trying to identify possible unreliable positives and unreliable negatives?. Arch Public Health.

[16] Hailey JR (2004). Pathology working group Chairperson’s report: lifetime study in rats conducted by the Ramazzini Foundation.

[17] EFSA (European Food Safety Authority) (2006). Opinion of the scientific panel on food additives, Flavourings, processing aids and materials in contact with food (AFC) on a request from the commission related to a new long-term carcinogenicity study on aspartame. EFSA J.

[18] Schoeb TR, McConnell EE, Juliana MM, Davis JK, Davidson MK, Lindsey JR (2009). Mycoplasma pulmonis and lymphoma in bioassays in rats. Vet Pathol.

[19] Caldwell JC, Jinot J, DeVoney D, Gift JS (2008). Evaluation of evidence for infection as a mode of action for induction of rat lymphoma. Environ Mol Mutagen.

[20] van Dongen JJ, Langerak AW, Brüggemann M, Evans PA, Hummel M, Lavender FL, Delabesse E, Davi F, Schuuring E, García-Sanz R, van Krieken JH, Droese J, González D, Bastard C, White HE, Spaargaren M, González M, Parreira A, Smith JL, Morgan GJ, Kneba M, Macintyre EA (2003). Design and standardization of PCR primers and protocols for detection of clonal immunoglobulin and T cell receptor gene recombinations in suspect lymphoproliferations: report of the BIOMED-2 concerted action BMH4-CT98-3936. Leukaemia.

[21] Willard-Mack CL, Elmore SA, Hall WC, Harleman J, Kuper CF, Losco P, Rehg JE, Rühl-Fehlert C, Ward JM, Weinstock D, Bradley A, Hosokawa S, Pearse G, Mahler BW, Herbert RA, Keenan CM (2019). Nonproliferative and Proliferative Lesions of the Rat and Mouse Hematolymphoid System. Toxicol Pathol.

[22] Tibaldi E, Gnudi F, Panzacchi S, Mandrioli D, Vornoli A, Manservigi M, Sgargi D, Falcioni L, Bua L, Belpoggi F (2020). Identification of aspartame-induced haematopoietic and lymphoid tumours in rats after lifetime treatment. Acta Histochem.

[23] Swerdlow SH, Campo E, Harris NL, Jaffe ES, Pileri SA, Stein H, Thiele J, Vardiman JW (2017). WHO classification of Tumours of Haematopoietic and lymphoid tissues Vol. 2. Revised Fourth Edition.

[24] Sylvetsky AC, Rother KI (2016). Trends in the consumption of low-calorie sweeteners. Physiol Behav.

[25] National Cancer Institute. SEER Database. Available at: http://seer.cancer.gov/.

[26] Andersen MS, Clubb DO (2013). Understanding and accounting for the costs of inaction. European Environment Agency. Late Lessons from Early Warnings.

[27] Lemen RA, Landrigan PJ (2017). Toward an asbestos ban in the United States. Int J Environ Res Public Health.

[28] Nicholson WJ, Landrigan PJ (1989). Quantitative assessment of lives lost due to delay in regulation of occupational exposure to benzene. Environ Health Perspect.

[29] Lim U, Subar AF, Mouw T, Hartge P, Morton LM, Stolzenberg-Solomon R, Campbell D, Hollenbeck AR, Schatzkin A (2006). Consumption of aspartame-containing beverages and incidence of hematopoietic and brain malignancies. Cancer Epidemiol Biomark Prev.

[30] Schernhammer ES, Bertrand KA, Birmann BM, Sampson S, Willett WC, Feskanich D (2012). Consumption of artificial sweetener- and sugar-containing soda and risk of lymphoma and leukemia in men and women. Am J Clin Nutr.

[31] Soffrritti M, Padovani M, Tibaldi E, Falcioni L, Manservisi F, Belpoggi F (2014). The carcinogenic effects of aspartame: the urgent need for regulatory re-evaluation. Am J Ind Med.

[32] IARC (International Agency for Research on Cancer), IARC Monographs Priorities Group. Advisory Group. 2019. Recommendations on priorities for the IARC Monographs. Lancet Oncol. 2019; .

